# Advancing Obstructive Airway Disease Treatment: Dual PDE3/4 Inhibition as a Therapeutic Strategy

**DOI:** 10.3390/cells14090659

**Published:** 2025-04-30

**Authors:** Rinzhin T. Sherpa, Cynthia J. Koziol-White, Reynold A. Panettieri

**Affiliations:** Rutgers Institute for Translational Medicine and Science, Rutgers University, 89 French Street, New Brunswick, NJ 08901, USA

**Keywords:** cAMP, PDE, ensifentrine, COPD, Asthma

## Abstract

Obstructive airway diseases, including asthma and chronic obstructive pulmonary disease (COPD), evoke significant global health concerns manifested by airway inflammation and obstruction. Despite their differing origins, shared pathophysiological features and responses to therapeutic interventions highlight common molecular mechanisms. Standard treatments include inhaled bronchodilators, with combination therapies offering enhanced symptom control. Cyclic AMP (cAMP) plays a crucial role in airway relaxation. Phosphodiesterase (PDE) decreases cAMP levels, thereby attenuating the relaxation of airway smooth muscle, making it a promising therapeutic target. The balance between cAMP production and degradation is essential for regulating airway tone and function. PDE inhibitors for the treatment of obstructive airway diseases have suffered challenges, with adverse side effects of prospective inhibitors causing clinical failures. Efforts to develop PDE inhibitors with an improved safety profile could prove to be beneficial as an add-on treatment for severe asthma and COPD. The recent FDA approval of Ensifentrine, a dual PDE3/4 inhibitor, can significantly advance COPD management by improving bronchodilation, reducing inflammation, and lowering exacerbation rates with favorable safety outcomes.

## 1. Introduction

Affecting millions of people worldwide, obstructive respiratory diseases are a global health problem and a major cause of chronic morbidity and mortality in the USA [1,2,3,4]. Asthma and chronic obstructive pulmonary disease (COPD) are marked by airway obstruction, and while these represent two different entities, they also manifest many parallels. While the root causes differ, similarities in airway obstruction and inflammation are observed in patients with asthma and those with COPD. Moreover, the beneficial response to similar therapeutic approaches in both diseases suggests similarities in molecular signaling pathways and pathophysiological characteristics [4].

**Figure 1:**
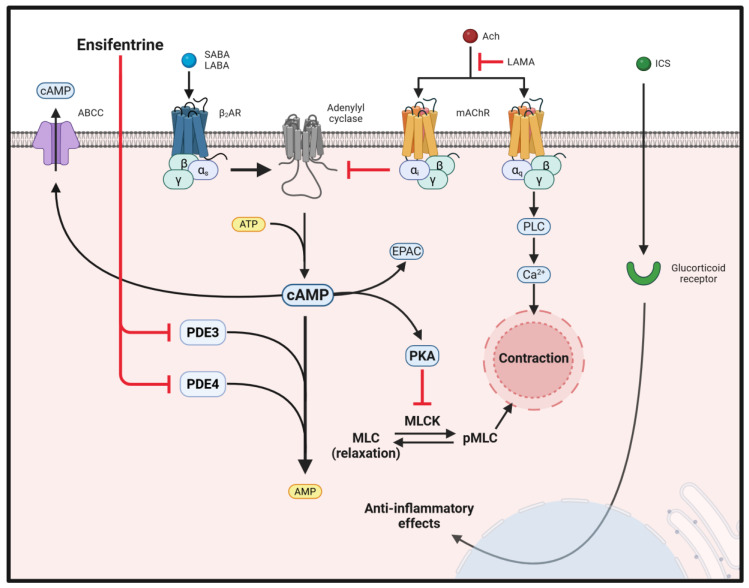
Obstructive airway disease medications and their interactions with cAMP signaling pathway. Cyclic AMP is a key messenger, with a critical role in airway relaxation, synthesized by adenylyl cyclase (AC) that are activated by Gαs and inhibited by Gαi. β_2_AR agonists (SABA and LABA) and muscarinic antagonists (LAMA) contribute to increasing cAMP levels. Some Muscarinic acetylcholine receptors (mAChR) isoforms, coupled to Gαq activate phospholipase C (PLC), stimulate increases in intracellular Ca^2+^ and promote contraction. Intracellular increase in cAMP activates protein kinase A (PKA), exchange protein activated by cAMP (Epac), and other effectors. cAMP signaling in airway smooth muscle cells ultimately leads to the phosphorylation of a myriad of downstream targets by PKA. Myosin light chain kinase (MLCK) regulates cell contractility by phosphorylating myosin light chains, which enables myosin to interact with actin, leading to muscle contraction. When MLCK is phosphorylated by PKA, there is a reduction in its ability to phosphorylate myosin light chains, thus decreasing muscle contraction. Phosphodiesterase isoforms (PDE) hydrolyze cAMP and brings down the intracellular cAMP levels. Using PDE inhibitors is a strategy to sustain increased cAMP levels to generate beneficial effects as seen in studies with Ensifentrine, a dual PDE3/4 inhibitor. In addition to the PDE-based reduction of cAMP, there ABCC1 (ATP-binding cassette [ABC] subfamily member C 1) membrane transporters that are vital for β-agonists evoked cAMP efflux to the extracellular environment. Another mainstay therapy is ICS, which acts through the glucocorticoid receptor and produces anti-inflammatory responses. Created in BioRender. Sherpa, RT (2025) https://biorender.com/z0tepql (accessed on 23 April 2025).

With asthma, exposure to triggers like allergens (pollen, dust mites, pet dander, etc.), irritants (smoke, chemical fumes, etc.), and other environmental factors can aggravate inflammation and cause airway constriction. Asthma exacerbations manifest symptoms of shortness of breath and chest tightness, and in severe cases have life-threatening episodes. Asthma medications work by relaxing the airways to improve ventilation, reduce wheezing, and alleviate other symptoms. In clinical settings, inhaled corticosteroids (ICS) and both short and long-acting β_2_ agonists (SABAs and LABAs, respectively) are the most used treatments in asthma management (Figure 1). Inhaled corticosteroids (ICS) act, in part, via the glucocorticoid receptor, which has profound anti-inflammatory effects, reducing inflammatory cell activation as well as suppressing pro-inflammatory mediator production and associated airway hyperresponsiveness (AHR) [5,6]. β_2_ agonists work by binding to the Beta-2 adrenergic receptors (β_2_AR), a stimulatory G protein-coupled receptor (GPCR), on the smooth muscle cells of the airways. This receptor binding triggers cAMP production and a subsequent cascade of events that leads to the relaxation of the smooth muscle, resulting in bronchodilation. For cases of moderate or persistent asthma, treatment options include LABAs, which provide prolonged bronchodilation and help prevent asthma symptoms for an extended period. Other options for asthma management include leukotriene modifiers, which target actions or production of leukotrienes, significant mediators in asthma-related inflammation, bronchoconstriction, and mucus production.

In COPD, patients have difficulty breathing because of a multitude of biological events, including mucosal buildup, inflammatory insults, airway constriction, and detrimental remodeling of the airways. Inflammatory mediators, such as cytokines, sensitize airway smooth muscle cells to acetylcholine and other contractile agonists, which induce smooth muscle cells to shorten or contract. Additionally, oxidative stress evoked by neutrophil-derived reactive oxygen species (ROS) damages lung tissue, breaking down alveolar structures crucial for gas exchange [7,8]. The multifaceted effects on pulmonary function trigger COPD patients to experience persistent symptoms that get worse over time. COPD is treated with some of the same medicines as asthma, such as inhaled bronchodilators, including long-acting muscarinic receptor antagonists (LAMAs), LABAs, and ICS [9,10,11]. Muscarinic antagonists disrupt the acetylcholine (Ach)-Gq-phospholipase C (PLC)-mediated increase in contraction-inducing intracellular Ca^2^⁺ to improve airflow in COPD patients by inhibiting airway smooth muscle contraction. In COPD patients who are unresponsive to treatment with dual bronchodilators and with no improvement in exacerbations, the use of a triple therapy of LAMA/LABA/ICS is beneficial in reducing exacerbations and mortality [12,13,14].

This combined use of LABAs with glucocorticoids provides long-lasting symptomatic relief and effective management of inflammatory responses. However, regular treatment with LABAs alone is associated with increased risks of severe exacerbations of asthma and death from asthma in a small subgroup of patients [15,16]. This has raised concerns regarding the safety of LABAs in asthma, but interestingly, in patients with concomitant COPD, these severe adverse reactions are absent [15].

While ICS is the most effective treatment for persistent asthma, in cases of severe disease, these drugs often fail to control symptoms fully. There are also concerns about the safety of ICS, such as increased incidence of respiratory infections and loss of bone density [17]. As with any disease space, it is always preferable to have a robust pipeline of pharmaceuticals that can be used to improve patient outcomes as well as standard of care. In the therapeutic landscape of obstructive airway disease, a comprehensive understanding of the pathophysiology and the intricate cellular signaling pathways operant in asthma and COPD should guide therapeutic options/strategies. Molecular entities with both bronchodilator and anti-inflammatory activity would give maximum benefit and need to be explored.

## 2. Cyclic AMP and PDEs

Cyclic AMP, a “second messenger”, modulates signal transduction pathways and influences cellular responses to external signals in a variety of different cell types [18,19]. Many physiological responses involve cAMP-dependent signaling, and studies have only further highlighted the importance and complexity of these signaling pathways [20]. Many GPCRs, like the β_2_AR, are expressed in airway smooth muscle (ASM) cells and rely on cAMP as their main second messenger. GPCRs modulate intracellular cAMP levels by coupling through Gs to induce cAMP synthesis through activation of adenylyl cyclases (AC) and/or through Gi proteins that block cAMP production [21,22]. In the airway, a rise in intracellular cAMP levels evokes relaxation of the ASM and inhibits inflammatory responses that contribute to the pathophysiology of asthma [19,23,24].

The relaxant effect of cAMP on ASM contractility is thought to act through the effector protein kinase A (PKA) [21]. PKA-based phosphorylation of numerous targets, including heat shock protein 20 (HSP20), leads to an impaired ability to promote myosin light chain (MLC) phosphorylation, which enables ASM to contract. Multiple pathways elicited by PKA activation, from effects on intracellular Ca^2+^ or reduced sensitivity to Ca^2+^ in ASM and mechanisms independent of MLC regulation, have been identified [19,25,26,27]. Furthermore, increased cAMP levels can affect mucociliary clearance directly through the activation and acceleration of ciliary motility, which depends on a balance between Ca^2+^ and cAMP levels, and indirectly by affecting allergic response or other inflammation [19,28].

As a family of enzymes, phosphodiesterases (PDEs) catalyze the hydrolysis of important second messengers, like cAMP and cyclic guanosine monophosphate (cGMP) [18]. Phosphodiesterases convert cAMP to 5′-AMP, which terminates the second messenger function of cAMP [29]. The PDE family consists of 11 groups that differ in structures, affinities for cAMP, sensitivities to inhibitors, and mechanisms of regulation [30] (Table 1). PDE4, PDE7, and PDE8 are cAMP-specific PDEs, whereas PDE1, PDE2, PDE3, PDE10, and PDE11 hydrolyze both cAMP and cGMP [21]. Transcriptomic data demonstrate the presence of all PDE isoenzymes, except PDE2, in human ASM cells derived from donors with asthma and fatal asthma [31].

As the predominant PDE isoenzyme in ASM, PDE3 inhibition increases cAMP and stimulates ASM relaxation [32,33]. PDE3 deficiency can reduce allergic airway inflammation and improve airway mucosal barrier function in allergic airway disease models [34]. PDE3 hydrolyses cAMP with relatively high affinity (*k*_m_ cAMP < 0.4 μM), and two genes have been identified for PDE3: *PDE3A* and *PDE3B*. PDE3A is expressed in vascular smooth muscle, airway smooth muscle, cardiac myocytes, platelets, oocytes, and B-lymphocytes. The PDE3B variant is highly expressed in adipocytes, hepatocytes, and spermatocytes and has also been detected in vascular smooth muscle cells, T-lymphocytes, and macrophages [35,36].

PDE4 isoenzyme represents the principal PDE isoenzyme expressed by most inflammatory cells of importance in the pathogenesis of asthma, including T cells, macrophages, eosinophils, and neutrophils, the ciliary epithelia, and in ASM cells, although its inhibition has not demonstrated acute bronchodilator effects in humans [28,37]. This observation is further supported by study results where the selective PDE3 inhibitor SKF94120 was more potent in inhibiting the contraction of human bronchi than the selective PDE4 inhibitor rolipram [38]. PDE4 has a relatively lower affinity for cAMP (*k*_m_ cAMP < 1–10 μM) and inhibition is associated with an anti-inflammatory effect [30,39]. The PDE4 family also has more members with four genes (*PDE4A*, *B*, *C*, *D*) combined with multiple splice variants that have a broad tissue distribution [40,41,42]. The PDE4D subfamily also appears to play a pivotal role in controlling cAMP degradation in human ASM cells [41]. Among the FDA-approved PDE inhibitors, only roflumilast, which targets PDE4, is used as an add-on therapy for COPD [43]. Adding roflumilast to ICS in asthma provided additional FEV_1_ improvement from baseline to 24 weeks [44,45]. There is experimental evidence that roflumilast stimulates both glucocorticoid receptor α (GRα) mRNA synthesis and GRα’s transcriptional activity in bronchial epithelial cells and enhances dexamethasone’s ability to suppress pro-inflammatory mediator production in a GRα-dependent manner [46]. However, roflumilast has been observed to cause gastrointestinal issues and weight-loss side effects, demoting it to a third-line medication. Studies have also shown that roflumilast improves sugar metabolism in obese patients and may decrease cardiovascular events in patients with COPD [47,48].

Based on the cAMP-elevating role of PDE inhibitors and their importance in airway biology, isoform-specific inhibitors have attracted significant interest with goals to achieve anti-inflammatory and pro-relaxant effects in the airway. PDE3 or 4 inhibitors are clinically available to treat various conditions, including COPD, but have cripplingly narrow therapeutic windows [43,49,50,51,52]. As a result of efforts to create a better mechanism to inhibit relevant PDEs, Ensifentrine (RPL554), which can inhibit both PDE3 and 4, has completed Phase III trials and was approved in June 2024 as a maintenance treatment for COPD [53,54,55,56]. Ensifentrine is a novel therapeutic option that targets bronchoconstriction and inflammatory responses with comparatively better tolerability and efficacy compared to previous PDE inhibitors [57].

**Table 1:** Various cAMP hydrolyzing PDE isoforms present in relevant cell types of the airway and effects of inhibition.

Cell Type	PDE Isoforms	Notes on Relevant Cellular Effects of Inhibition	Ref
**Airway Epithelium**	PDE1	Inhibition blockaded lipopolysaccharide-endotoxin (LPS)-mediated biosynthesis of interleukin (IL)-6.	[58,59,60,61,62,63]
	PDE3	Inhibition of PDE3 leads to activation of cystic fibrosis transmembrane conductance regulator (CFTR). Blocking PDE3 activity also abolished the effect of LPS on IL-6 and attenuated TNF-α production.	[58,62,64]
	PDE4	Inhibition induces repressive effect on IL-6, IL-8 production and a dual, biphasic (excitatory/inhibitory) effect on TNF-α secretion. Also induces increased production of PGE_2_.	[58,59,60,61,62,65]
	PDE7	PDE7 inhibition synergizes with PDE4 inhibition to suppress inflammatory signaling.	[60,66,67]
**Airway Smooth Muscle (ASM)**	PDE1	Inhibition increases ciliary beat frequency and angle in lung airway cell.	[31,68]
	PDE3	Blocking PDE3 activity promotes bronchodilation by relaxing smooth muscle.	[66,69,70]
	PDE4	Inhibition reduces ASM hyperreactivity and inflammation by stopping pro-inflammatory signaling.	[31,69]
	PDE7	Airway reactivity and contractility are decreased after PDE7 inhibition.	[66,71]
	PDE8	Inhibition of PDE isoform enhances isoproterenol induced reduction in cell proliferation.	[31]
**Goblet Cells**	PDE4	Inhibition reduces mucus hypersecretion by downregulating MUC5AC expression.	[72]
**Submucosal Glands**	PDE3	PDE3 inhibition augments CFTR-dependent submucosal gland secretion.	[73,74]
	PDE4	Inhibition of PDE4 stimulates elevated saliva production.	[75]
**Eosinophils**	PDE4	Functions such as the release of inflammatory granule constituents, chemotaxis, cytokines and superoxide generation are inhibited by blocking PDE4	[65,66,76,77]
	PDE7		[66]
**Neutrophils**	PDE3	Inhibition reduces neutrophil chemotaxis and activation.	[76,77,78,79]
	PDE4	Inhibition suppresses neutrophil degranulation and function (Leukotriene B4 and reactive oxygen species (ROS) synthesis).	[65,76,78]
**Macrophages**	PDE1	Inhibition decreases macrophage-mediated inflammation and oxidative stress.	[50,66,76,79,80]
	PDE3	Inhibition can play an anti-inflammatory role in allergic airway inflammation.	[36]
	PDE4	PDE4 inhibitors reduce generation of pro-inflammatory cytokine, TNF-α, from macrophages in the presence of PDE3 inhibitor. Inhibition also potentiates chemokine expression elicited by forskolin or Prostaglandin E2 (PGE_2_).	[61,76,81]
	PDE7	May work in concert with PDE4 inhibition to further suppress macrophage-driven inflammation.	[66,82]
**T lymphocytes**	PDE3	Inhibition affects T cell activation and proliferation, potentially modulating immune responses in COPD.	[65,66,76,78,83]
	PDE4	Inhibition suppresses T cell proliferation, activation and cytokine production (IL-4, IL-5, and IFN-γ synthesis), reducing airway inflammation.	[76]
	PDE7	PDE7 inhibition decreases proliferation, IL-12 expression, and acts synergistically with PDE4 inhibition.	[66,69,78,84]
	PDE8	Inhibition suppresses attachment of T effector cells to endothelial cells.	[81]

## 3. Ensifentrine, a Dual PDE3 and 4 Inhibitor

Phosphodiesterase inhibitors have potential as a treatment for obstructive airway diseases. Theophylline, a non-specific phosphodiesterase inhibitor with bronchodilator and anti-inflammatory effects, has been used in the management of asthma [85,86,87]. However, theophylline exhibits adverse side-effects, and this encourages a search for effective and safer PDE inhibitors [88].

Recently, a novel dual inhibitor of PDE3 and PDE4, Ensifentrine (C_26_H_31_N_5_O_4_) from Verona Pharma, was approved by the FDA as a maintenance treatment for patients with COPD [56,89]. The dual inhibition of PDE3 and 4 by ensifentrine has been shown to relax airway smooth muscle as well as suppress inflammatory signals [56]. In addition to its relaxant properties, *in vitro* studies with ensifentrine show a stimulatory effect on the cystic fibrosis transmembrane conductance regulator (CFTR), which can benefit mucociliary clearance of the respiratory tract [90]. Due to these beneficial effects seen in COPD, ensifentrine is being considered for the treatment of asthma as well as cystic fibrosis (Table 2).

The parent molecule of ensifentrine is trequinsin, a dual PDE3/4 inhibitor with long-lasting bronchodilator properties in airway smooth muscle compared to other PDE inhibitors [91]. Ensifentrine is a moderately potent PDE3 inhibitor (IC50 = 0.4 nM) and a weak PDE4 inhibitor (IC50 = 1479 nM) [54]. A variety of *in vitro* and *in vivo* assays of relevance to airway function have been used to test the effects and efficacy of ensifentrine. In guinea pig tracheal preparations, ensifentrine produced relaxation of airway smooth muscle induced by spasmogens (e.g., histamine, carbachol), whereas the efficacy of the other bronchodilators varied according to the contractile stimulus used [92]. *In vitro* studies using isolated human bronchi showed that ensifentrine inhibited bronchial contraction induced by electrical field stimulation, relaxed bronchi precontracted with acetylcholine, and abolished bronchial contraction induced by histamine [93].

*In vitro* ensifentrine, in a concentration-dependent manner, inhibited lipopolysaccharide-induced tumor necrosis factor α (TNF-α) release from human monocytes and proliferation of human mononuclear cells to phytohemagglutinin [54]. Anti-inflammatory activity of ensifentrine was further reflected *in vivo* where it significantly inhibited eosinophil recruitment following antigen challenge in ovalbumin-sensitized guinea pigs. In response to histamine challenge, ensifentrine significantly inhibited histamine-induced bronchoconstriction as well as plasma protein extravasation in the trachea, signifying both pro-relaxant and anti-inflammatory effects of ensifentrine [54,94]. Based on the concept of combination treatments that have been adopted in the treatment of asthma and COPD, it is valuable to have information on potential synergistic effects between ensifentrine and other therapeutics. Analysis of combination effects shows that ensifentrine synergizes with muscarinic receptor antagonists, atropine or glycopyrrolate, to produce a greater relaxant effect than either compound alone [92,93].

## 4. Clinical Trials

While encouraged by the evidence of ensifentrine eliciting both bronchodilator and anti-inflammatory activities, critical hurdles exist to assess its safety profile, which has slowed development of other PDE inhibitor candidates. To answer these questions, trials were conducted to test the safety and efficacy of inhaled ensifentrine in healthy participants and in patients with asthma or COPD (Table 2).

Franciosi *et al.* reported a series of exploratory clinical studies conducted from 2009 to 2013, where ensifentrine showed substantial bronchodilator, broncho-protective, and anti-inflammatory effects with minimal side effects [95]. The trial population consisted of healthy participants, patients with mild-to-moderate asthma, and patients with mild-to-moderate COPD. Ensifentrine produced a bronchodilatory response as well as a broad anti-inflammatory effect, as measured by changes in lipopolysaccharide-induced recruitment of sputum neutrophils. As a bronchodilator, ensifentrine had a rapid and significant effect comparable to salbutamol, a β_2_AR agonist clinically used as a rescue inhaler for acute relief. In asthmatics, repeated ensifentrine doses also maintained bronchodilator effects [95].

In 2015 (NCT02542254) and 2017 (NCT03028142), two phase II studies reported by Singh *et al.* explored the ability of ensifentrine to increase the bronchodilator effects of the β_2_AR agonist salbutamol, and muscarinic antagonists ipratropium bromide or tiotropium bromide in patients with stable moderate-to-severe COPD [96]. Overall, the two studies demonstrate that ensifentrine combination treatment provided additional benefits on spirometric measurement of forced expiratory volume in 1 second (FEV_1_). FEV_1_ measures the volume of air exhaled forcefully in the first second of a breath and provides a measure of overall air flow in the respiratory system. Reduced FEV_1_ values, which can be rated from 0 (At Risk) to 4 (Very Severe COPD), indicate severity of airway obstruction and are a hallmark of obstructive lung diseases [97]. In obstructive lung diseases, a variety of factors, including inflammation, swelling, mucus overproduction, and loss of tissue elasticity, reduce the volume of air that can be expelled. Monitoring FEV_1_ in patients over time can reveal progression of obstructive disease and the effectiveness of treatment. Moreover, Ensifentrine was well tolerated in the patient populations where it was administered alone or in combination with bronchodilators [96,98].

As a dual PDE3/4 inhibitor, it is logical to assume that ensifentrine could cause adverse effects seen with previous PDE inhibitors targeting those specific isoforms. Milrinone, a PDE3 inhibitor, is used in cases of acute heart failure to induce positive inotropy and is administered through an intravenous route (IV). Nebulized drugs achieve much less systemic effects and absorption than the IV route and could be a factor as to why no cardiovascular effects were seen with nebulized ensifentrine, even though it is a potent PDE3 inhibitor. No gastrointestinal side effects, usually seen with orally administered PDE4 inhibitors, were reported at any dose of ensifentrine. These short-term phase II studies with positive results in bronchodilation and improved airway conductance by ensifentrine provided further incentive to proceed with longer-term studies to its sustained efficacy, safety profile, and potential benefits in reducing symptoms and exacerbations and improving quality of life in patients with obstructive airway diseases.

A Dose Ranging Study (twice daily nebulized 0.75, 1.5, 3, or 6 mg) of ensifentrine in COPD patients showed a dose-response up to 3 mg, which then plateaus at a higher doses of 6 mg (NCT03443414; EudraCT 2016–005205-40) [99]. Delivery modalities such as Dry Powder Inhaler (DPI) and Metered-Dose Inhaler (MDI) for Ensifentrine have been tested (NCT04027439 and NCT04091360) in Phase II trials and have met all of the primary and secondary endpoints. While the nebulizer formulation has been approved for the maintenance treatment of COPD, the investigation and successful results of DPI and MDI formulations indicates potential in development of more convenient alternatives. In determining delivery methods, factors related to drug delivery efficacy, patient capabilities, device usability, formulation compatibility, and cost are all considered [100]. While inhalers are generally portable and offer fast administration, COPD patients often face difficulties using inhalers due to poor inspiratory flow. Moreover, nebulizer-based delivery provides effective lung deposition with consistent dosing [100,101,102].

Two Phase III trials, ENHANCE-1 and -2 (“Ensifentrine as a Novel inhAled Nebulized COPD therapy”) (NCT04535986, EudraCT identifier 2020-002086-34 & NCT04542057, EudraCT identifier 2020-002069-32), evaluating nebulized ensifentrine for the maintenance treatment of COPD, showed that this drug induces an improvement in lung function and significantly reduces the rate and risk of COPD exacerbations [56,103]. The patient groups in ENHANCE-1 and ENHANCE-2 were current or former smokers with a mean patient age of 65 years. The mean post-bronchodilator FEV_1_ was <52% predicted normal and the mean smoking history was >41 pack-years. The study conducted 24 or 48 weeks of ensifentrine treatment and evaluation of lung function, symptoms, quality of life, and exacerbations.

Historically, clinical trials of pharmaceutical interventions in COPD have included improvement in trough (predose or pre-bronchodilator) FEV1, which reflects the efficacy over 12 to 24 h of 100–150 mL, although there is no definite evidence or consensus in the minimal clinically important difference (MCID) [104,105]. MCID has been defined as ‘‘the smallest difference in score in the domain of interest which patients perceive as beneficial and which would mandate, in the absence of troublesome side effects and excessive costs, a change in the patient’s management” [106]. MCID trough FEV_1_ also varies in terms of treatment. A review of clinical trials provides MCID trough FEV_1_ values for LABA (Salmeterol; 78–107 mL), LAMA (Ipratropium Bromide; 121 mL), ICS (45 mL), and PDE4 inhibitors (50–100 mL), with combination treatments achieving higher FEV_1_ values [104]. In the clinical trials constituting the development of ensifentrine in COPD, FEV_1_ area under the curve at 0–12 h (AUC_0–12h_), which evaluates the full effect profile ensifentrine with changes in efficacy over time, has been designated as a primary outcome.

Patients in the ENHANCE-1 (69% of total) and ENHANCE-2 (55% of total) had background therapy with LAMA, LABA, ICS/LAMA, or ICS/LABA throughout the trials. In ENHANCE-1, the least squares mean (LSM) change from baseline in FEV_1_ AUC_0–12 h_ post dose at 12 weeks was 61 mL in the twice-daily ensifentrine compared with -26 mL in the placebo arm [56,103]. The St. George’s Respiratory Questionnaire (SGRQ) focuses on the measurement of impact on overall health, daily life, and perceived well-being in patients with obstructive airways disease. Based on empirical data and interviews with patients, a mean change score of 4 units is associated with slightly efficacious treatment, 8 units for moderately efficacious change, and 12 units for very efficacious treatment [107]. In both ENHANCE-1 and -2 trials, the responder rate, which was defined as an improvement in the score of ≥4, was higher in the ensifentrine arm compared with the placebo arm. Furthermore, ensifentrine treatment reduced the frequency of exacerbations and extended the time to first exacerbation among patients with moderate to severe exacerbations.

The ENHANCE-1 and ENHANCE-2 trials showed that ensifentrine was well tolerated in patients with moderate to severe COPD [56,108]. The rates of adverse events associated with ensifentrine were similar to those observed with placebo with pooled data from both trials groups reporting treatment-emergent adverse events (TEAEs) (36.8% vs. 35.9%) and serious TEAEs (6.2% vs. 6.3%) in ensifentrine vs. placebo after 24 weeks of treatment. ENHANCE-1 and ENHANCE-2 both demonstrated reductions in the rate of moderate to severe COPD exacerbations, with ENHANCE-2 showing a 43% reduction compared to 36% in ENHANCE-1. Notably, ENHANCE-1 included a subgroup of patients treated for 48 weeks, providing valuable long-term efficacy and safety data. In ENHANCE-1, 84.5% of randomized patients completed the trial, whereas ENHANCE-2 had a completion rate of 77.3%.

In conclusion, the results of both the ENHANCE-1 and ENHANCE-2 trials were overwhelmingly positive, validating the clinical development of ensifentrine as a promising treatment for COPD. The desirable outcomes in reducing exacerbations, improving lung function, and demonstrating a favorable safety profile contributed to the FDA’s approval of ensifentrine for the maintenance treatment of COPD. These trials provided robust evidence that ensifentrine can effectively manage COPD symptoms and reduce the frequency of exacerbations, offering a new therapeutic option among the limited treatment mechanisms available for COPD patients.

## 5. Discussion

By targeting both PDE3 and PDE4, ensifentrine enhances airway relaxation and reduces inflammation, improving outcomes for patients with asthma and COPD. Based on these clinical findings, ensifentrine can be considered a valuable addition to enhance the overall management of obstructive airway diseases and provide greater relief to these patients. However, long-term studies of safety, efficacy, and comparative effectiveness comparing existing treatments of ensifentrine need to be carried out. These studies may determine whether patients may develop resistance to this class of inhibitors over a longer time frame and how ensifentrine functions in combination with other therapies over the same period.

Further research is also required to drill down the mechanisms by which ensifentrine mediates its potent effects. PKA and EPAC are the major downstream effectors of cAMP. PKA plays a central role in the relaxation of airway smooth muscle cells by inhibiting MLCK and promoting myosin light chain dephosphorylation, hyperpolarizing the membrane, and reducing calcium influx, which decreases contractile force generation [24,109]. A recent study demonstrated that EPAC activation in lung endothelial cells stimulated with extracellular histones led to superior barrier-enhancing and protective properties, and it caused dramatic decreases in several inflammatory markers, including VCAM-1, ICAM-1, and pro-inflammatory cytokines [110]. Al Matni *et al.* suggest that EPAC activation is involved in mediating ensifentrine’s protective effects on primary lung endothelial cells and alveolar epithelial cell lines [18]. Moreover, with ensifentrine’s selectivity for PDE3 versus PDE4, and keeping in mind the relative expression of PDE3 and PDE4 in the cells of the airways, it would be valuable to determine which PDE isoenzyme is most responsible for each effect. While PDE inhibitors prevent the hydrolysis of cAMP, a study by Cao *et al.* provides an intriguing perspective on cAMP regulation by examining the role of cAMP efflux by ABCC1 (ATP-binding cassette (ABC) subfamily member C1) in human ASM [111]. The study showed that ABCC1 promotes bronchodilator-stimulated cAMP leakage out of ASM, and this cAMP efflux prevents enhanced ASM relaxation. It is prudent to also place importance on cAMP efflux mechanisms based on their direct impact on reducing cAMP levels in ASM, which diminishes airway relaxation and serve as an additional cAMP regulatory mechanism, besides PDE activity, that modulates efficacy of bronchodilators [111].

The regulation of cAMP in the cells is complex and recent evidence shows nanodomains of cyclic nucleotide signaling where certain isoforms of PDEs are more critical than others. It is this subcellular compartmentalization of cyclic nucleotide signaling that enables a single cell to respond discretely to multiple extracellular and intracellular signals. These signaling nanodomains can be held together by scaffolding proteins like A-kinase anchoring proteins (AKAPs) or arise when PKA undergoes liquid-liquid phase separation (LLPS) to form biomolecular condensates enriched in cAMP and PKA activity [20,112]. Drugs/mechanisms that allow modulation of not only total cellular cAMP but rather compartmentalized cAMP and signaling could be the next promising approach. Such targeted approaches, at a subcellular level, could potentially avoid adverse reactions that have been apparent with general PDE inhibitors. The development of dual PDE3/4 inhibitors like ensifentrine represents a significant advancement in the treatment of obstructive airway diseases, offering both bronchodilatory and anti-inflammatory benefits with a promising safety profile.

## Figures and Tables

**Table 2:** Clinical trials conducted to study the safety and efficacy of Ensifentrine (RPL554) in obstructive airway diseases. Sourced from www.clinicaltrials.gov.

Phases	NCT Number	Study Title	No. of Patients Enrolled	Interventions	Start Date	Primary Completion
PHASE1	NCT02307162	SAD/MAD Study of a New Formulation of Nebulized RPL554 in Healthy Subjects and COPD Subjects	112	Ensifentrine|Placebo	December 2014	July 2015
PHASE2	NCT02427165	Comparison of RPL554 With Placebo and Salbutamol in Asthmatic Patients	29	Ensifentrine|Salbutamol|Placebo	April 2015	November 2015
	NCT02542254	The Effects of RPL554 on Top of Standard COPD Reliever Medications	36	Ensifentrine|Salbutamol|Ipratropium|Placebo	October 2015	December 2015
	NCT03028142	The Effects of RPL554 in Addition to Tiotropium in COPD Patients	30	Ensifentrine|Placeboin addition to tiotropium	January 2017	August 2017
	NCT02919995	A Study of RPL554 in Patients With Cystic Fibrosis	10	Ensifentrine|Placebo	February 2017	November 2017
	NCT03443414	Dose Ranging Study of RPL554 in Chronic Obstructive Pulmonary Disease (COPD) Patients	405	Ensifentrine|Placebo	June 2017	January 2018
	NCT03673670	Bronchodilator Effect of RPL554 Administered in Addition to Tiotropium/Olodaterol in Patients With COPD	79	Ensifentrine|Placebo|Tiotropium/olodaterol (Respimat)	July 2018	November 2018
	NCT04027439	Study Evaluating 5 Doses of RPL554 and Placebo in COPD Patients Via a Dry Powder Inhaler	37	Ensifentrine|Placebo	December 2018	May 2019
	NCT04091360	A Study of RPL554 Drug Administered by Metered Dose Inhaler to Treat Chronic Obstructive Pulmonary Disease	40	Ensifentrine|Placebo	April 2019	December 2020
	NCT03937479	Study Investigating the Effect of 4 Doses of RPL554 Given in Addition to Tiotropium to Patients With COPD	416	Ensifentrine|Placebo in addition to tiotropiuin	May 2019	November 2019
	NCT04527471	Pilot Study of Ensifentrine or Placebo Delivered Via pMDI in Hospitalized Patients With COVID-19	45	Ensifentrine|Placebo	September 2020	February 2021
	NCT05270525	Effect of Ensifentrine on Sputum Markers of Inflammation in COPD	50	Ensifentrine|Placebo	May 2022	December 2026
	NCT06559150	A Phase II Study of Ensifentrine in Non-Cystic Fibrosis Bronchiectasis	180	Ensifentrine|Placebo	September 2024	September 2026
PHASE3	NCT04542057	A Phase 3 Trial to Evaluate the Safety and Efficacy of Ensifentrine in Patients With COPD	790	Ensifentrine|Placebo	September 2020	May 2022
	NCT04535986	A Phase 3 Clinical Trial to Evaluate the Safety and Efficacy of Ensifentrine in Patients With COPD	763	Ensifentrine|Placebo	September 2020	September 2022
	NCT06460493	Effect of Ensifentrine Treatment on CAT Score	20	Ensifentrine	June 2024	November 2024

## Data Availability

Data sharing is not applicable.

## Abbreviations

The following abbreviations are used in this manuscript:

ABCC1	ATP-binding cassette [ABC] subfamily member C1
AC	Adenylyl cyclase
Ach	Acetylcholine
AHR	Airway Hyperresponsiveness
AKAP	A-kinase anchoring protein
ASM	Airway smooth muscle
β_2_AR	Beta-2 adrenergic receptor
cAMP	3′,5′-cyclic adenosine monophosphate
cGMP	3′,5′-cyclic guanosine monophosphate
COPD	Chronic obstructive pulmonary disease
Epac	Exchange protein activated by cAMP
FEV	Forced expiratory volume
GPCR	G protein-coupled receptor
GRα	Glucocorticoid receptor α
HSP	Heat shock protein
ICS	Inhaled corticosteroids
LABA	Long-Acting Beta-Agonists
LAMA	Long-Acting Muscarinic antagonists
mAChR	Muscarinic acetylcholine receptor
MLC	Myosin light chain
MLCK	Myosin light chain kinase
PDE	Phosphodiesterase
PKA	Protein kinase A
PLC	Phospholipase C
ROS	Reactive oxygen species
SABA	Short-Acting Beta-Agonists
